# Epigenetics in Ascending Thoracic Aortic Aneurysm and Dissection

**DOI:** 10.1055/s-0038-1639610

**Published:** 2018-07-27

**Authors:** Adeline Boileau, Mark E. Lindsay, Jean-Baptiste Michel, Yvan Devaux

**Affiliations:** 1 Cardiovascular Research Unit, Luxembourg Institute of Health, Luxembourg, Luxembourg; 2 Department of Pediatrics, Massachusetts General Hospital, Harvard Medical School, Boston, Massachusetts; 3 UMRS 1148, INSERM, Paris 7-Denis Diderot University, Hôpital Xavier Bichat, Paris, France

**Keywords:** noncoding RNAs, histone acetylation, DNA methylation

## Abstract

Thoracic aortic aneurysm (TAA) is an asymptomatic and progressive dilatation of the thoracic aorta. Ascending aortic dissection (AAD) is an acute intraparietal tear, occurring or not on a pre-existing dilatation. AAD is a condition associated with a poor prognosis and a high mortality rate. TAA and AAD share common etiology as monogenic diseases linked to transforming growth factor β signaling pathway, extracellular matrix defect, or smooth muscle cell protein mutations. They feature a complex pathogenesis including loss of smooth muscle cells, altered phenotype, and extracellular matrix degradation in aortic media layer. A better knowledge of the mechanisms responsible for TAA progression and AAD occurrence is needed to improve healthcare, nowadays mainly consisting of aortic open surgery or endovascular replacement. Recent breakthrough discoveries allowed a deeper characterization of the mechanisms of gene regulation. Since alteration in gene expression has been linked to TAA and AAD, it is conceivable that a better knowledge of the causes of this alteration may lead to novel theranostic approaches. In this review article, the authors will focus on epigenetic regulation of gene expression, including the role of histone methylation and acetylation, deoxyribonucleic acid methylation, and noncoding ribonucleic acids in the pathogenesis of TAA and AAD. They will provide a translational perspective, presenting recent data that motivate the evaluation of the potential of epigenetics to diagnose TAA and prevent AAD.

Aortic progressive dilation (aneurysm) is a potentially life-threatening condition that puts patients at risk of aortic dissection or rupture, a devastating clinical event with high mortality. Most aneurysms are painless and cause no symptomatology to alert the patient or clinician to the associated risk. Unfortunately, the hidden nature of progressive extracellular matrix (ECM) defect (with or without dilation) means that often the diagnosis is only made at the time of an aortic dissection. Thousands of deaths annually can be attributed to aortic aneurysms and dissections. 1

Epigenetics can be defined as stable heritable traits independent of changes in deoxyribonucleic acid (DNA) sequence. In many cardiovascular disorders, significant epigenetic modifications have been shown to affect disease development or progression. Work has progressed most rapidly in cardiomyocytic lineages where intensive effort has gone into understanding transcriptional networks at the epigenetic level. 2 In cardiomyocytic lineages, multiple noncoding ribonucleic acids (RNAs) have been identified that coordinate protein complexes to direct promoter modification or other remote transcriptional enhancers. Despite significant progress in the identification of the genetic basis of aortic aneurysms, the full range of chromatin modifications that affect disease progression is just beginning to be investigated. This review will cover the known literature regarding epigenetic control of ascending thoracic aortic aneurysm (TAA) and ascending aortic dissection (AAD), and highlight opportunities for future research in this important topic.

## Thoracic Aortic Aneurysm and Dissection: Pathophysiology

Dilating remodeling of arterial tissues is defined as the structural consequence of dysregulated biological activities controlling the ECM and cell homeostasis within the wall. Aneurysms, regardless of their localization, are characterized by breakdown of the ECM, and aortic smooth muscle cell (aSMC) loss, leading to progressive dilation of the arteries, thinning of the medial layer, and, eventually leading to rupture of the arterial wall. Risk factors for aortic aneurysm are dependent on the regional propensity of the affected segment. Descending thoracic aortic aneurysm (DTAA) demonstrates a pathology characterized by atheroma, aSMC disarray and apoptosis, intense phagocytosis, and intraluminal thrombus usually observed in abdominal aortic aneurysms (AAA). Enzymatic degradation of the aortic ECM leads to structural weakening, dilation, and eventually rupture if left untreated. 3 A large proportion of patients with DTAA also coexpress AAA. In contrast, the ascending thoracic aorta is relatively protected. Instead, TAAs have a pathologic appearance characterized by a leukocyte-independent “medial degeneration” in which medial aSMCs play the major role.

Thoracic aortic aneurysm, a progressive dilation of the thoracic ascending aorta, is a chronic pathology involving an imbalance between proteolytic degradation of the ECM and wall compensating resistance to proteolytic injury, 4 involving aSMC adaptive capacities (phenotypic switches, mainly from contractile to synthetic state). TAA associates with highly penetrant genetic conditions that segregate with single genes, 5 but can also have a degenerative etiology in elderly patients, or be associated with bicuspid aortic valves, in which a specific hemodynamic disorder could play an important role. 6 Whatever the etiology, hemodynamics, including high shear stress and arterial blood pressure, play a direct or indirect role in progressive dilation or acute tear.

The identity of genes mediating TAA often represents ECM components or regulators and genes required for the contractile function of aSMC. Increased expression of matrix-degrading enzymes, mucoid accumulation, and aSMC apoptosis are commonly noted in most forms of aneurysmal disease. Disruption of structural ECM components such as collagens and elastin is a commonly accepted mechanism of aneurysm pathogenesis. It comes as no surprise, therefore, that mutations that directly influence the ECM components can cause aneurysm. Examples include mutations in ELN, 7 encoding elastin, COL3A1, 8 encoding collagen type 3-α1, microfibril-associated protein 5, 9 or FBN1, encoding fibrilin1, and responsible for Marfan syndrome. 10 Additionally, mutations in genes encoding proteins required for elastogenesis and collagen metabolism such as EFEMP2 11 and LOX 12 promote TAA. In these disorders, altered assembly or decreased expression of matrix components results in weakness of the aortic media.

In addition to genetic changes that directly alter ECM components, human mutations have been discovered that interfere with vascular smooth muscle-mediated aortic homeostasis in genes encoding members of the smooth muscle contraction apparatus, such as MYH11, 13 encoding smooth muscle myosin and ACTA2, 14 encoding α-smooth muscle actin, and PRKG-1, 15 encoding type I cGMP-dependent protein kinase. Mutations are commonly encountered in genes encoding the various members of the canonical transforming growth factor β (TGF-β) signaling cascade such as TGF-β receptors TGFBR1 and TGFBR2, 16 their cognate ligands TGF-β2, 17 TGF-β3, 18 and SMAD3, an intracellular effector of TGF-β signaling. 19 Mutations in TGF-β receptors are responsible for the Loeys–Dietz syndrome. 16

Aortic dissection is an acute intramural rupture, which can occur in a normal or moderately dilated aorta, 20 without compensatory mechanisms. TAA and AAD correspond respectively to a progressive or acute loss of the ability of the arterial wall to withstand the arterial blood pressure.

TAA and AAD share common etiologies, including monogenic diseases in young patients. 13 21 They also feature common pathological signatures, including aSMC cell disappearance, areas of mucoid degeneration, 22 and degradation of collagen and elastic fibers. 23 Overall, TAA and AAD develop through interactions between numerous factors, including genetics—from mutations causing Mendelian traits to genetic susceptibility, environmental factors such as specific hemodynamic conditions, 24 and aSMC phenotypic changes, some of which are induced by epigenetic modifications.

## Epigenetics: General Concepts

**Fig. 1:**
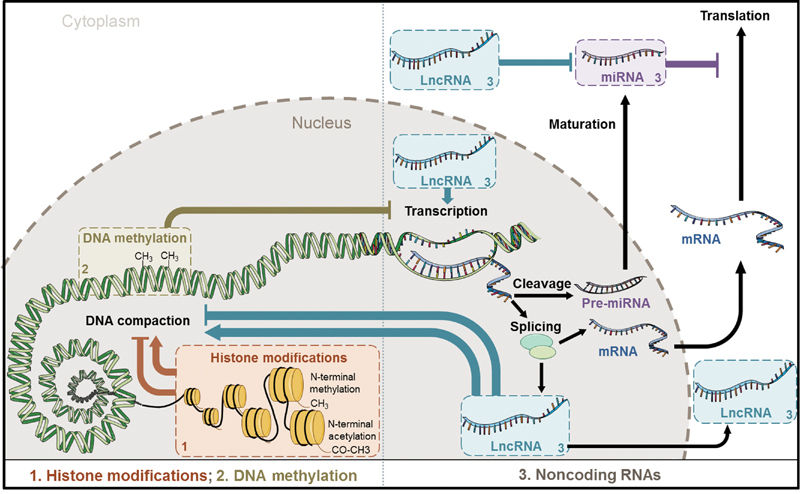
Overview of epigenetic mechanisms. Epigenetic modifications include (1) modifications of DNA-associated histone proteins, (2) DNA methylation, and (3) noncoding RNAs. Abbreviations: DNA, deoxyribonucleic acid; LncRNA, long noncoding RNA; mRNA, messenger RNA; miRNA, microRNA; RNA, ribonucleic acid.

As displayed in Fig. 1 , epigenetic modifications encompass different mechanisms: modifications of DNA-associated histone proteins, DNA methylation, and noncoding RNAs-mediated modifications. 25 These mechanisms target either DNA molecules, transcriptional machinery, or transcription products, resulting in modulation of gene expression and consequent protein synthesis.

Histones are DNA-associated proteins responsible for chromatin compaction. They are prone to modifications such as acetylation, methylation, phosphorylation, adenosine diphosphate-ribosylation, and sumoylation that generally occur on specific amino acid residues (arginine and lysine) of their N-extremity. 26 Histone acetylation is associated with chromatin loosening and activates transcription, while histone methylation can either activate or repress transcription. 27

Performed by DNA methyltransferases, 28 DNA methylation forms a covalent but reversible bond between a methyl group and the DNA base cytosine, resulting in the production of 5-methylcytosine, sometimes considered as the “fifth base” of DNA. DNA methylation is commonly associated with repression of transcription. Methylated DNA can interact with other epigenetics changes as histone-code modifications. 29

Noncoding RNAs are defined as RNA molecules lacking protein-coding potential. They are generally classified according to their size: small noncoding RNAs contain less than 200 nucleotides, while long noncoding RNAs (lncRNAs) contain at least 200 base pairs. Different types of small noncoding RNAs have been characterized, among which 20-nucleotides-long microRNAs (miRNAs) predominate. 30 MiRNAs possess a seed sequence (6 to 8 nucleotides in length), homologous with the 3′ untranslated region of their target genes. The binding of miRNAs to their target gene through base complementarity results in repression of the expression of the target gene. 31 This repression occurs through messenger RNA (mRNA) degradation when the complementarity between miRNAs and target genes is complete and through inhibition of translation when this complementarity is partial. 32 Importantly, several miRNAs can bind the same mRNA and a single miRNA can bind different mRNAs, attesting for the complexity of gene regulation by miRNAs.

The mechanisms of gene regulation by lncRNAs are more complex and diverse than miRNAs. LncRNAs can act as signal, decoy, guide, scaffold, or enhancer. 33 Some lncRNAs mediate their effects by guiding or recruiting proteinaceous complexes to initiate transcription. 34 The lncRNA Xist modulates gene expression by regulation of imprinting. 35 The lncRNA named CDR1-AS is circular and acts as a miRNA-sponge, preventing the binding of miRNAs to target genes, hence favoring their expression. 36 LncRNAs can also compete with other genes or RNA transcripts, acting as competitive endogenous RNA. 37

The presence of miRNAs in the circulation was revealed almost a decade ago 38 and a plethora of studies on the potential of miRNAs for use as biomarkers of cardiovascular disease rapidly emerged. 39 The biomarker potential of circulating lncRNAs has been more recently evidenced. 33 40 41 Circular RNAs, which are more stable than their linear counterparts due to resistance to exonuclease degradation, also appear to possess an interesting biomarker potential. 42 43

## Epigenetics in Thoracic Aortic Aneurysm Disease

### Histone Modifications

Experimental studies reported an activation of TGF-β in aneurysms related to Marfan syndrome. 44 TGF-β signaling in syndromic and nonsyndromic aneurysmal diseases was investigated in different types of aneurysmal ascending aortic specimens. The TGF-β1 mRNA was not significantly changed. In contrast, the amounts of TGF-β1 protein retained within and released by an aneurysmal media layer were greater than for a healthy aortic media layer. This observation fitted with the observed increase in LTBP-1 mRNA and protein, 45 associated with the increase in ECM protein turnover reported in TAA. 46 This was associated with higher levels of Smad2 mRNA, phosphorylated Smad2 protein, and nucleus translocation in the ascending aortic wall from all types of aneurysm. Activation of TGF-β pathway was correlated with the degree of elastic fiber fragmentation. Surprisingly, there was no consistent colocalization between the nuclear location of phospho-Smad2 and extracellular TGF-β staining. This first study highlights independent dysregulations of TGF-β retention and Smad2 signaling in TAA whatever their etiologies, suggesting a non-TGF-β dependent activation and nuclear translocation of Smad2 in TAA. 45

In a second study performed on human aortic samples, including TAAs of different etiologies and normal aortas from which tissue extracts and aSMCs and fibroblasts were obtained in primary cultures, it was observed that all types of TAA share a complex dysregulation of Smad2 signaling, independent of TGF-β1 in TAA-derived aSMCs. 47 The Smad2 dysregulation was characterized by an aSMC-specific (not seen in fibroblasts) heritable activation and overexpression of Smad2, compared with normal aortas. The cell specificity and heritability of this overexpression of Smad2 suggested the implication of an epigenetic control. Using chromatin immunoprecipitation assay, the authors showed that Smad2 overexpression in TAA involves increases in H3K9/14 acetylation and H3K4 methylation. 47 These results demonstrated the heritability, cell specificity, and independence with regard to TGF-β1 and genetic backgrounds of the Smad2 dysregulation in thoracic aneurysms and the involvement of epigenetic mechanisms regulating histone marks in this process.

In a third study, the histone-modifying enzymes, transcription factors, and cofactors responsible for Smad2 promoter activation in aSMC from TAA patients were explored to understand the mechanisms regulating Smad2 overexpression. It was shown that Smad2 promoter activation is driven by the recruitment of a multipartner complex, including the transcription factor p53 and histone acetyltransferases. 48 Remarkably, the transcriptional regulatory network of the Smad2 promoter was dramatically altered in human aneurysmal aSMCs in vitro and in situ with a switch from Myc-dependent repression of Smad2 in normal vessel to a p53-dependent constitutive activation of Smad2 in aneurysms. Furthermore, histone acetyltransferases p300 and P300/CBP-associated proteins played a major role in Smad2 promoter activation by acting on histone acetylation and p53 recruitment. 48 These results provided evidence for a major role of p53 and the complex composed of histone acetyltransferases p300 and p300/CBP-associated proteins in Smad2 activation in human aneurysmal aSMCs.

**Fig. 2:**
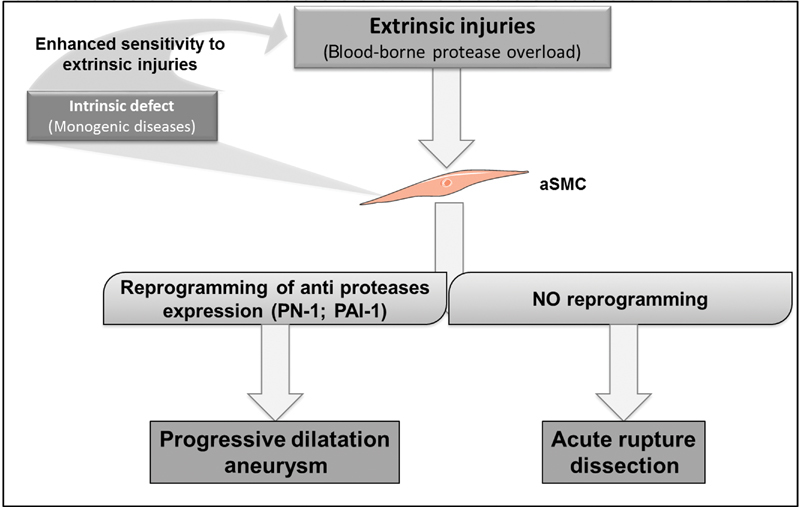
Schematic representation of the differential biology between chronic dilation of the aorta (aneurysm) and acute intraparietal rupture (dissection). The progressive development of chromatin remodeling in aortic smooth muscle cells (aSMC) in response to small dilation or matrix proteolytic injury could reduce the risk of acute rupture. Abbreviations: PN-1, protease nexin-1, PAI-1, plasminogen activator inhibitor-1.

In a fourth study, protease nexin-1 (PN-1) and plasminogen activator inhibitor-1 (PAI-1), both inhibitors of serine-protease were overexpressed in medial tissue extracts and primary aSMCs from TAA compared with acute dissections of ascending aortic and healthy aorta. 49 Furthermore, TGF-β increased PN-1 expression in control but not in aneurysmal aSMCs. PN-1 and PAI-1 overexpression by aneurysmal aSMCs was associated with increased Smad2 binding on their respective gene promoters. This phenomenon was restricted to aneurysms and not observed in acute dissections. 49 Thus, epigenetically regulated PN-1 overexpression promotes development of an antiproteolytic aSMC phenotype which might favor progressive aneurysmal dilation. Absence of this counter regulation may lead to acute wall rupture (dissection, Fig. 2 ).

### DNA Methylation

A genome-wide DNA methylation study comparing aortic tissues from patients with two different etiologies of TAA, bicuspid and tricuspid aortic valves, revealed that several genes involved in cardiovascular development were differentially methylated, 50 suggesting that DNA methylation may be involved in TAA development and dissection.

### MicroRNAs

Although the functional contribution of miRNAs to the development of TAA and progression toward dissection is still poorly characterized, some associations between miRNAs expression levels, TAA and AAD, have been reported ( Table 1 ).

**Table 1:** Differentially expressed miRNAs in aortic tissues from patients with TAA or AAD compared with controls

	AAD aortic tissue			TAA aortic tissue		
ID	Expression versus control	Technology	Reference	Expression versus control	Technology	Reference
miR-1				↓	Microarray, PCR	52 54
miR-15a	↓	Microarray	51	↑	Microarray	53
miR-21				↑ ; ↓	Microarray; PCR	52 53 54
miR-21 a				↑	Microarray	53 54
miR-22	↓	Microarray, PCR	51	↓	Microarray	53 a
miR-25				↑	Microarray	53
miR-29a	↓	Microarray	51	↓	Microarray, PCR	52 54
miR-29b				↑	Microarray; PCR	53 57
miR-30c a				↓	PCR	54
miR-125a-3p				↓	Microarray	53
miR-126–3p				↑	Microarray; PCR	53 a 54
miR-128				↑	Microarray	53
miR-133a	↓	Microarray	51	↓	Microarray, PCR	52 53 54
miR-133b	↓	Microarray	51	↓	Microarray	53
miR-138–1 a	↑	Microarray	51	↑	Microarray	53
miR-142–5p				↑	Microarray	53
miR-143	↓	Microarray, PCR	51	↓	PCR	67
miR-145	↓	Microarray, PCR	51	↓ ; ↑	Microarray; PCR	53 54 66 67
miR-146b-5p				↑	Microarray	53 54
miR-155				↓	PCR	54
miR-183 a	↑	Microarray, PCR	51	↑	Microarray	53
miR-204				↓	PCR	54
miR-422a				↑	Microarray	53
miR-433	↑	Microarray, PCR	51			
miR-486–5p				↓ ; ↑	Microarray; PCR	53 54
miR-487b				↑	Microarray	53
miR-491–3p	↑	Microarray, PCR	51	↑	Microarray	53 b
miR-553	↑	Microarray, PCR	51			
miR-638				↓	Microarray	53 b 54
miR-940				↓	Microarray	53
miR-193a-3p; miR-768–5p; miR-886–5p; miR-30e; miR-195; miR-101; miR-140–5p; miR-744	↓	Microarray	51	↑	Microarray	53 a
miR-193a-5p	↓	Microarray	51	↓	Microarray	53 b

Abbreviations: AAD, ascending aortic dissection; PCR, polymerase chain reaction; TAA, thoracic aortic aneurysm

a Only in males.

b Only in females.

↑: upregulated in disease versus control.

↓: downregulated in disease versus control.

Underlined: miRNAs differentially expressed in TAA and AAD.

#### MicroRNA Profiles

Technological developments of high-throughput approaches such as microarray facilitated the characterization of disease-associated miRNA profiles.

Using microarray and quantitative polymerase chain reaction (PCR), a panel of four up-regulated and three down-regulated miRNAs was identified in six aortic tissues from AAD patients compared with six control subjects 51 ( Table 1 ). In another study, four miRNAs were down-regulated in aortic tissues from TAA patients compared with control subjects, and their expression levels were inversely correlated with aortic diameter. 52 Also with microarray, a set of 16 miRNAs was found to be regulated in 10 nonfamilial and nonsyndromic aneurysmal ascending aorta compared with 10 control aorta. 53 Interestingly, gender-associated differences in miRNA profiles were observed in this study. Yet, no validation by PCR was reported. More recently, 6 up-regulated and 2 down-regulated miRNAs were observed in 11 aortic tissues from TAA patients compared with 8 control tissues, using quantitative PCR. 54 Noteworthy, even though two different diseases (AAD and TAA) were investigated in these four studies, 51 52 53 54 at least nine miRNAs (miR-22/-29a/-133a/-133b/-138–1*/-145/-183*/-193a-5p/-491–3p) showed similar expression patterns ( Table 1 ). Considering that TAA and AAD display similar features, these studies support the involvement of miRNAs in TAA and AAD pathogenesis. However, miR-1/-30c-2/-145/-204/-331–3p were up-regulated in human aortic tissue samples from patients with TAA compared with DTAA. 55 Since TAA and DTAA possess different clinical features and etiologies, such differences of miRNA profiles strengthen the concept that miRNAs may play specific roles in TAA and DTAA pathogenesis.

Investigation of the functional role of miRNAs in TAA and AAD remains difficult in human aortic tissues in situ. Gender-associated differences need to be considered. The use of primary human aSMC and experimental models in mice allowed a better characterization of the mechanisms of action of miRNAs, as detailed hereafter.

#### MiR-29 Family

MiR-29 family contains three members, miR-29a/-29b/-29c, all involved in fibrosis. 56 MiR-29b was highly expressed in human aSMC compared with endothelial cells and monocytes. 57 Its expression was higher in aorta from TAA patients compared with healthy controls, whereas expression of miR-29a and miR-29c was comparable between the two groups. 57 In humans, however, miR-29a expression was decreased during TAA formation. 52 54 In mice, miR-29 family members were overexpressed in aorta from fibulin-4 deficient animals. 57 MiR-29b was also overexpressed in angiotensin II-infused mice and its silencing with locked nucleic acid (LNA)-modified antisense oligonucleotides inhibited angiotensin II-triggered aortic dilation. 57 MiR-29b was up-regulated in the ascending aorta of Marfan Fbn1 ^(C1039G/ + )^ mice and its inhibition with LNA-modified antisense oligonucleotides prevented aneurysm development, reduced apoptosis in aortic wall, and increased ECM components synthesis. 58

In vitro, miR-29b induced aSMC migration by inhibiting DNA methyltransferase 3 that negatively regulates matrix metalloproteinases 2 and 9 expression. 59 MiR-29b directly targets the genes encoding the antiapoptotic proteins MCL1 (a member of the BCL2 family of apoptosis regulators) 60 and KLF4 (Kruppel-like factor 4). 61 KLF4 is a transcription factor involved in aSMC proliferation and is activated by Sp1. 62 MiR-29 family members also down-regulate the expression of several genes coding for ECM proteins such as collagens 1a1, 1a2, 3a1, eln1, fbn1, 63 and Adamts7. 64 TGF-β1 stimulation increased miR-29b expression in aSMC from Fbn ^C1039G/+^ mice but not from wild-type mice. 58 This stimulation reduced nuclear factor kappa B (NFκB) activity and, since miR-29b expression is inhibited by NFκB in ascending aorta of young Fbn ^C1039G/+^ mice, the authors proposed that up-regulation of miR-29b expression by TGF-β1 may involve the NFκB pathway. 58

These studies support a therapeutic potential for miR-29b for the prevention of TAA expansion, acting through modulation of aSMC synthetic phenotypic switch and ECM composition. The cross-talk between miR-29b and TGF-β is consistent with a role for miR-29b in the pathogenesis of conditions due to TGF-β pathway alteration, not limited to TAA and AAD. The observation that TGF-β impacts miR-29b expression suggests that TAA and AAD pathogenesis is controlled by complex epigenetic mechanisms that remain to be fully deciphered.

Intriguingly, in aorta from young Fbn ^C1039G/+^ mice, losartan administration reduced aneurysm formation and miR-29b expression. 58 Furthermore, expression of miR-29 family members was increased in aorta of aged mice compared with young mice. 57 These observations may explain, at least in part, the heterogeneity of results obtained from human studies, mostly negative. Medication and age are certainly confounding factors that need to be taken into account in future clinical studies.

#### MiR-143/-145 Family

MicroRNA-143 and miR-145 are expressed from the same bicistronic precursor. 65 Both miRNAs were down-regulated in aorta from AAD patients compared with controls 51 and miR-145 was up-regulated in TAA compared with DTAA. 55 In another study, miR-145 expression was higher in TAA tissues compared with control tissues and was positively correlated with collagen III levels and aortic diameter. 66 Elsewhere, miR-143/-145 expression was deficient in aortic tissues from TAA patients compared with controls. 67 These discrepancies may be attributed to technical bias (use of microarray or PCR to measure miRNAs) or differences between patient cohorts and disease etiology.

In mice, miR-143 and miR-145 were enriched in aorta compared with other organs. 67 Homozygous miR-143/-145 knocked-out mice showed altered aortic structure compared with the wild-types, as characterized by diminution of medial thickness, and aSMC dedifferentiation and migration toward the intima. 67

In vitro, miR-145 has been proposed as a phenotypic marker of vascular smooth muscle cells (vSMC). 68 MiR-143/-145 appear to play an important role in vSMC phenotypic switch since their inhibition increased cell migration and decreased proliferation. 67 Furthermore, miR-143/-145 up-regulation increased the expression of vSMC differentiation markers. 68 The effects of miR-145 on vSMC are mediated by modulation of the TGF-β pathway 66 and targeting of KLF4 and KLF5. 65 Using cocultured vascular endothelial cells and vSMC, a communication involving exosomes containing miR-143/-145 and transferring from endothelial cells to vSMC could be demonstrated. 69

Despite some heterogeneity observed in human studies, miR-143/-145 family is apparently deeply involved in the regulation of aSMC biology and ECM composition. Since aSMC phenotypic switch and ECM alterations are critical in aortic media dysfunction, miR-143 and miR-145 may play important roles in TAA and AAD pathogenesis that could be interesting for therapeutic purposes.

#### MicroRNAs in aSMC Phenotypic Switch

**Fig. 3:**
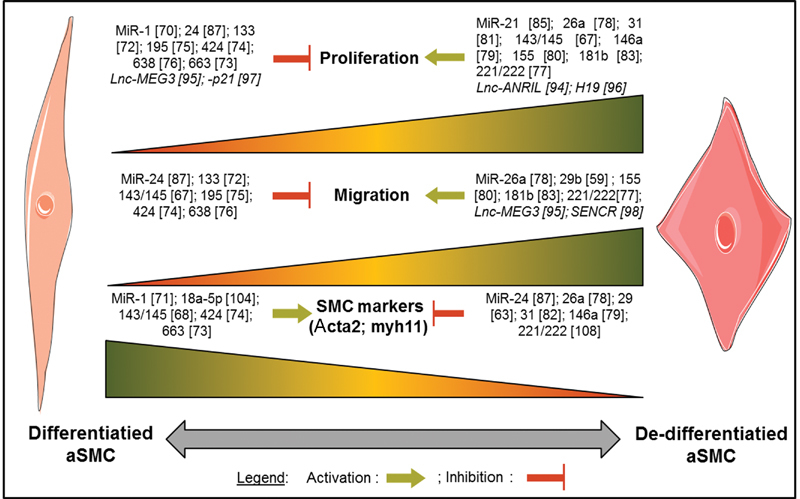
Involvement of microribonucleic acids (miRNAs) and long noncoding RNAs (lncRNAs) in aortic smooth muscle cell (aSMC) phenotypic switch.

Due to the importance of aSMC phenotypic switch in the pathogenesis of TAA and AAD, the following chapter focuses on miRNAs ( Fig. 3 ) and their target genes ( Table 2 ) known to regulate this switch.

**Table 2:** MicroRNAs target genes modulating aSMC phenotypic switch

ID	Target genes	Effect in aSMC	Reference
miR-1	Klf4	Induces aSMC markers expression: acta2, tagln	71
miR-133	Sp1	Inhibits cnn1; tagln2; acta2 and sfr; induces myh11 expression	72
miR-143/145	Klf4, CamkII-δ, Klf5	Induces acta2; cnn1; myh11; col3 expression and TGF-B	65 66 68
miR-146a	Klf4	Inhibits acta2; induces NFKB phosphorylation	79 102
miR-181b	Eln	Inhibits p21/p27; induces CDK4 and cyclin D1. Induces erk1/2 and jnk/jnk phosphorylation	83 103
miR-18a-5p		Induces cnn1; acta2 expression	104
miR-21	Sp1; c-Ski	Inhibits myh11 expression	105 106
miR-221/222	p27(Kip1), p57(Kip2), and c-Kit	Inhibits aSMC markers: acta2, cnn, and tagln expression	77 107 108
miR-24	HMOX-1; PDGFRB and c-Myc	Inhibits HMGB1; erk, and akt activation and inhibits aSMC markers acta2 and ccn1 expression	86 87 109
miR-26a		Inhibits myh11; acta2 ; smad1 and smad 4	78
miR-29b	Mcl-1 ; Klf4 ; Col1a1 ; col1a2 ; col3a1 ; eln ; fbn1 ; adamts7	Inhibits col1a2 ; col3a1 ; eln ; adamts7 expression	57 60 61 63 64
miR-31	CREG; lats2	Inhibits acta2 expression	81 82
miR-424		Induces acta2, myh11 and cnn1 expression	74
miR-663	JunB and Myl9	Induces acta2; myh11, tagln, ccn1	73

Abbreviations: aSMC, aortic smooth muscle cell; CREG, cellular repressor of E1A-stimulated gene; MicroRNA, microribonucleic acid.

- *MicroRNAs promoting aSMC differentiation* . MiR-1 expression was induced by myocardin in aSMC, 70 targeted KLF4, and induced aSMC differentiation from embryonic stem cells. 71 MiR-133 repressed Sp1 (which activates KLF4 62 ), resulting in maintenance of aSMC in a differentiated status. 72 MiR-133 also inhibited aSMC migration, acta2 and srf expression, and was associated with an increase in myh11 expression. 72 MiR-663 and miR-424 induced the expression of aSMC differentiation markers (acta2; myh11) and decreased aSMC migration. 73 74 MiR-195 repressed proliferation, migration, and protein secretion of aSMC. 75 MiR-638 inhibited aSMC proliferation and migration. 76
- *MicroRNAs promoting aSMC dedifferentiation* . MiR-221 and miR-222 are two close homologous miRNAs sharing the same seed sequence. They are highly expressed in aSMC, inducing not only their proliferation but also their migration, and inhibiting their apoptosis, presumably through modulation of their target genes p27 (Kip1), p57 (Kip2), and c-Kit. 77 Interestingly, miR-221/-222 had opposite effects on endothelial cells compared with aSMC. 77 MiR-26a targets the TGF-β/BMP (bone morphogenic protein) pathway, thereby inhibiting aSMC apoptosis and differentiation, and activating proliferation and migration. 78 MiR-146a and miR-155 induced aSMC migration and repressed apoptosis. 79 80 MiR-31 was strongly expressed in aSMC and induced their proliferation, 81 probably through modulation of cellular repressor of E1A-stimulated genes and acta2. 82 MiR-181b induced proliferation, and migration of aSMC through the PI3K and MAPK pathways. 83
- *MicroRNAs promoting phenotype switch* . The role of miR-21 in aSMC phenotype switch is less clear since it appears to be able to promote aSMC differentiation 84 as well as proliferation 85 which is considered as a feature of dedifferentiation. Likewise, miR-24 inhibited aSMC proliferation and migration 86 but repressed the expression of contractile gene markers. 87

Overall, the differential expression of multiple miRNAs in aneurysmal aortic tissues, as well as their functional involvement in aSMC biology and ECM composition, strongly supports their role in the pathogenesis of TAA and AAD. The challenge resides in the differentiation between simple bystanders and miRNAs that actively contribute to the disease, the latter category containing the most interesting therapeutic candidates. MiR-29b appears to be such a good candidate.

To our knowledge, the biomarker potential of circulating miRNAs has not been studied in the context of TAA. The potential use of circulating miRNAs as markers of AAA and aneurysm growth has only recently been reported. 88 Being able to predict dissection with a simple blood test, based on miRNA measurements, for instance, would represent a major achievement.

#### Long Noncoding RNAs

Contrarily to miRNAs, a few studies have addressed the association between lncRNAs and TAA. The lncRNA AK056155 was up-regulated in serum and aortic tissue samples from patients with Loeys–Dietz syndrome compared with controls. 89 Since these patients generally develop TAA, it is conceivable that AK056155 is also associated with TAA. However, all patients with Loeys–Dietz syndrome enrolled in this study had AAA, precluding evaluation of the association between AK056155 and TAA. Another lncRNA, named HIF1α-AS1 for its localization on the antisense strand of the gene coding the hypoxia inducible factor 1 α, was strongly up-regulated in serum of patients with thoraco-AAA. 90 HIF1α-AS1 down-regulation in palmitic acid-treated aSMC inhibited apoptosis and this effect was associated with a decrease in caspase-3, -8, and bcl 2 expression. 90 91

Mice lacking the lncRNA GAS5 display a thickening of the aortic media. 92 GAS5 is expressed in aortic wall, endothelial cells, and aSMC. In aSMC, its down-regulation increased viability and proliferation and lowered expression of aSMC markers. 92 Interestingly, GAS5 may act as paracrine mediator since conditioned medium obtained from endothelial cells overexpressing GAS5 induced a decrease in proliferation and migration of aSMC. 92 Knocking out the lncRNA RNCR3 in mice lowered aSMC proliferation, an effect attributed to a competition for miR-185–5p binding. 93

In vitro experiments revealed that, like miRNAs, lncRNAs affect aSMC proliferation and migration ( Fig. 3 ). The lncRNA ANRIL (CDKN2B antisense RNA 1) induced aSMC proliferation and up-regulated some genes involved in TAA such as eln and col3a1. 94 MEG3 reduced proliferation and increased migration of aSMC, effects accompanied by p53 and mmp2 overexpression. 95 H19 induced aSMC proliferation, an effect mediated by miR-675. 96 LincRNA-p21 inhibits aSMC proliferation and apoptosis by regulating p53 activity. 97 SENCR is highly expressed in aSMC and its down-regulation repressed the expression of aSMC markers and increased cell migration. 98

The role of lncRNAs in TAA is only emerging. The observation that some lncRNAs are present in blood supports a potential use as biomarkers. A deeper characterization of the functional involvement and biomarker potential of lncRNAs in TAA and AAD is warranted.

## Conclusion and Future Directions

Genetic alterations critically account for the development of TAA and the progression toward dissection. However, other etiologies of TAA or AAD such as degenerative TAA or AAD occurring on medium-sized aorta are unrelated to any known DNA polymorphism. In this context, epigenetic modifications, which do not change DNA sequence, may also trigger, or at least contribute to, TAA and AAD. Several lines of evidence strongly support a causative role of epigenetic modifications and noncoding RNAs in the pathogenesis of TAA and AAD.

Since epigenetic modifications may occur under the influence of environmental changes such as hemodynamic modifications, it would be interesting to address whether chromatin remodeling mediated by histone modifications is related to the hemodynamic modifications featured in TAA. Indeed, the dilation (increased radius, *r* ) observed in TAA is associated with a decreased media thickness ( *h* ), leading to a dramatic increase in wall tension ( *T* ) at a constant blood pressure ( *P* ), according to the Laplace's law ( *T* = *P* . *r* /2 *h* ). The nuclear envelope is mechanically coupled to mechanotransduction through aSMC adhesion to matrix, coupling of integrins to actin, intermediate filaments (tensegrity 99 ) and linkers of nucleoskeleton to cytoskeleton. 100 101 It has been proposed that the mechanical environment impacts the chromatin status of aSMC, thereby controlling vascular gene expression and function. 87 It is, therefore, likely that the increased wall tension in TAA leading to altered mechanotransduction signaling between the matrix and the nucleus will impact chromatin remodeling in aSMC, an issue that has not been addressed. In addition, since chromatin remodeling is associated with chronic progressive dilation but not with acute intraparietal rupture (dissection without important dilation), chromatin remodeling in aSMC could be a hallmark of TAA as compared with dissecting aortas.

The impact of noncoding RNAs on TAA and AAD pathogenesis is far from being well characterized. Increasing evidences show that both short and lncRNAs interact to regulate chromatin remodeling, aSMC phenotype, and ECM composition, sometimes through mediation of cell–cell communication.

Since miRNAs and lncRNAs are present in the blood, it is conceivable that they may emanate, actively or passively, from the diseased vascular wall and inform about its stability or propensity to rupture. The possibility that noncoding RNAs could be used as biomarkers of TAA and dissection is attractive and remains to be tested.

Additional research is warranted to reach a better knowledge of the epigenetic mechanisms gearing TAA development and aneurysm rupture. Several studies reported here showed the involvement of noncoding RNAs in TAA and dissection. However, their role in the pathogenesis of these diseases is only partly understood. Increasing interest is observed toward DNA methylation and histone modification. New insights into their role in TAA and dissection pathogenesis are expected to emerge in the upcoming years. Since epigenetic changes can be modulated, this may lead to novel tools for better diagnosis and treatment of these severe conditions.

## References

[1] Benjamin E J Blaha M J Chiuve S E Heart Disease and Stroke Statistics-2017 Update: a report from the American Heart Association Circulation 2017 135 10 e146 e603 28122885 10.1161/CIR.0000000000000485 PMC5408160

[2] Bruneau B G Epigenetic regulation of the cardiovascular system: introduction to a review series Circ Res 2010 107 03 324 326 20689071 10.1161/RES.0b013e3181f17dfe PMC2930886

[3] Kim J B Kim K Lindsay M E Risk of rupture or dissection in descending thoracic aortic aneurysm Circulation 2015 132 17 1620 1629 26338955 10.1161/CIRCULATIONAHA.114.015177

[4] Jondeau G Michel J B Boileau C The translational science of Marfan syndrome Heart 2011 97 15 1206 1214 21742617 10.1136/hrt.2010.212100

[5] Vapnik J S Kim J B Isselbacher E M Characteristics and outcomes of ascending versus descending thoracic aortic aneurysms Am J Cardiol 2016 117 10 1683 1690 27015890 10.1016/j.amjcard.2016.02.048

[6] Michelena H I Della Corte A Prakash S K Milewicz D M Evangelista A Enriquez-Sarano M Bicuspid aortic valve aortopathy in adults: incidence, etiology, and clinical significance Int J Cardiol 2015 201 400 407 26310986 10.1016/j.ijcard.2015.08.106

[7] Szabo Z Crepeau M W Mitchell A L Aortic aneurysmal disease and cutis laxa caused by defects in the elastin gene J Med Genet 2006 43 03 255 258 16085695 10.1136/jmg.2005.034157 PMC2563239

[8] Superti-Furga A Gugler E Gitzelmann R Steinmann B Ehlers-Danlos syndrome type IV: a multi-exon deletion in one of the two COL3A1 alleles affecting structure, stability, and processing of type III procollagen J Biol Chem 1988 263 13 6226 6232 2834369

[9] Barbier M Gross M S Aubart M MFAP5 loss-of-function mutations underscore the involvement of matrix alteration in the pathogenesis of familial thoracic aortic aneurysms and dissections Am J Hum Genet 2014 95 06 736 743 25434006 10.1016/j.ajhg.2014.10.018 PMC4259978

[10] Magenis R E Maslen C L Smith L Allen L Sakai L Y Localization of the fibrillin (FBN) gene to chromosome 15, band q21.1 Genomics 1991 11 02 346 351 1769651 10.1016/0888-7543(91)90142-2

[11] Dasouki M Markova D Garola R Compound heterozygous mutations in fibulin-4 causing neonatal lethal pulmonary artery occlusion, aortic aneurysm, arachnodactyly, and mild cutis laxa Am J Med Genet A 2007 143A 22 2635 2641 17937443 10.1002/ajmg.a.31980

[12] Guo D C Regalado E S Gong L LOX mutations predispose to thoracic aortic aneurysms and dissections Circ Res 2016 118 06 928 934 26838787 10.1161/CIRCRESAHA.115.307130 PMC4839295

[13] Zhu L Vranckx R Khau Van Kien P Mutations in myosin heavy chain 11 cause a syndrome associating thoracic aortic aneurysm/aortic dissection and patent ductus arteriosus Nat Genet 2006 38 03 343 349 16444274 10.1038/ng1721

[14] Guo D C Pannu H Tran-Fadulu V Mutations in smooth muscle alpha-actin (ACTA2) lead to thoracic aortic aneurysms and dissections Nat Genet 2007 39 12 1488 1493 17994018 10.1038/ng.2007.6

[15] Guo D C Regalado E Casteel D E Recurrent gain-of-function mutation in PRKG1 causes thoracic aortic aneurysms and acute aortic dissections Am J Hum Genet 2013 93 02 398 404 23910461 10.1016/j.ajhg.2013.06.019 PMC3738837

[16] Loeys B L Chen J Neptune E R A syndrome of altered cardiovascular, craniofacial, neurocognitive and skeletal development caused by mutations in TGFBR1 or TGFBR2 Nat Genet 2005 37 03 275 281 15731757 10.1038/ng1511

[17] Lindsay M E Schepers D Bolar N A Loss-of-function mutations in TGFB2 cause a syndromic presentation of thoracic aortic aneurysm Nat Genet 2012 44 08 922 927 22772368 10.1038/ng.2349 PMC3616632

[18] Bertoli-Avella A M Gillis E Morisaki H Mutations in a TGF-β ligand, TGFB3, cause syndromic aortic aneurysms and dissections J Am Coll Cardiol 2015 65 13 1324 1336 25835445 10.1016/j.jacc.2015.01.040 PMC4380321

[19] van de Laar I M Oldenburg R A Pals G Mutations in SMAD3 cause a syndromic form of aortic aneurysms and dissections with early-onset osteoarthritis Nat Genet 2011 43 02 121 126 21217753 10.1038/ng.744

[20] LeMaire S A Russell L Epidemiology of thoracic aortic dissection Nat Rev Cardiol 2011 8 02 103 113 21173794 10.1038/nrcardio.2010.187

[21] Boileau C Guo D C Hanna N TGFB2 mutations cause familial thoracic aortic aneurysms and dissections associated with mild systemic features of Marfan syndrome Nat Genet 2012 44 08 916 921 22772371 10.1038/ng.2348 PMC4033668

[22] de Figueiredo Borges L Martelli H Fabre M Touat Z Jondeau G Michel J B Histopathology of an iliac aneurysm in a case of Menkes disease Pediatr Dev Pathol 2010 13 03 247 251 19522551 10.2350/08-08-0516.1

[23] de Figueiredo Borges L Jaldin R G Dias R R Stolf N A Michel J B Gutierrez P S Collagen is reduced and disrupted in human aneurysms and dissections of ascending aorta Hum Pathol 2008 39 03 437 443 18261628 10.1016/j.humpath.2007.08.003

[24] Bäck M Gasser T C Michel J B Caligiuri G Biomechanical factors in the biology of aortic wall and aortic valve diseases Cardiovasc Res 2013 99 02 232 241 23459103 10.1093/cvr/cvt040 PMC3695745

[25] Egger G Liang G Aparicio A Jones P A Epigenetics in human disease and prospects for epigenetic therapy Nature 2004 429(6990):457 463 15164071 10.1038/nature02625

[26] Lee K K Workman J L Histone acetyltransferase complexes: one size doesn't fit all Nat Rev Mol Cell Biol 2007 8 04 284 295 17380162 10.1038/nrm2145

[27] Geiman T M Robertson K D Chromatin remodeling, histone modifications, and DNA methylation-how does it all fit together? J Cell Biochem 2002 87 02 117 125 12244565 10.1002/jcb.10286

[28] Bestor T H The DNA methyltransferases of mammals Hum Mol Genet 2000 9 16 2395 2402 11005794 10.1093/hmg/9.16.2395

[29] Jin B Li Y Robertson K D DNA methylation: superior or subordinate in the epigenetic hierarchy? Genes Cancer 2011 2 06 607 617 21941617 10.1177/1947601910393957 PMC3174260

[30] Devaux Y Transcriptome of blood cells as a reservoir of cardiovascular biomarkers Biochim Biophys Acta 2017 1864 01 209 216 27836747 10.1016/j.bbamcr.2016.11.005

[31] He L Hannon G J MicroRNAs: small RNAs with a big role in gene regulation Nat Rev Genet 2004 5 07 522 531 15211354 10.1038/nrg1379

[32] Goretti E Wagner D R Devaux Y Regulation of endothelial progenitor cell function by micrornas Minerva Cardioangiol 2013 61 06 591 604 24253453

[33] Devaux Y Zangrando J Schroen B Long noncoding RNAs in cardiac development and ageing Nat Rev Cardiol 2015 12 07 415 425 25855606 10.1038/nrcardio.2015.55

[34] Uchida S Dimmeler S Long noncoding RNAs in cardiovascular diseases Circ Res 2015 116 04 737 750 25677520 10.1161/CIRCRESAHA.116.302521

[35] Engreitz J M Pandya-Jones A McDonel P The Xist lncRNA exploits three-dimensional genome architecture to spread across the X chromosome Science 2013 341(6147):1.237973E6 10.1126/science.1237973 PMC3778663 23828888

[36] Hansen T B Jensen T I Clausen B H Natural RNA circles function as efficient microRNA sponges Nature 2013 495(7441):384 388 23446346 10.1038/nature11993

[37] Guo G Kang Q Zhu X A long noncoding RNA critically regulates Bcr-Abl-mediated cellular transformation by acting as a competitive endogenous RNA Oncogene 2015 34 14 1768 1779 24837367 10.1038/onc.2014.131

[38] Mitchell P S Parkin R K Kroh E M Circulating microRNAs as stable blood-based markers for cancer detection Proc Natl Acad Sci U S A 2008 105 30 10513 10518 18663219 10.1073/pnas.0804549105 PMC2492472

[39] Goretti E Wagner D R Devaux Y miRNAs as biomarkers of myocardial infarction: a step forward towards personalized medicine? Trends Mol Med 2014 20 12 716 725 25457620 10.1016/j.molmed.2014.10.006

[40] Kumarswamy R Bauters C Volkmann I Circulating long noncoding RNA, LIPCAR, predicts survival in patients with heart failure Circ Res 2014 114 10 1569 1575 24663402 10.1161/CIRCRESAHA.114.303915

[41] Vausort M Wagner D R Devaux Y Long noncoding RNAs in patients with acute myocardial infarction Circ Res 2014 115 07 668 677 25035150 10.1161/CIRCRESAHA.115.303836

[42] Devaux Y Creemers E E Boon R A Circular RNAs in heart failure Eur J Heart Fail 2017 19 06 701 709 28345158 10.1002/ejhf.801

[43] Vausort M Salgado-Somoza A Zhang L Myocardial infarction-associated circular RNA predicting left ventricular dysfunction J Am Coll Cardiol 2016 68 11 1247 1248 27609688 10.1016/j.jacc.2016.06.040

[44] Lindsay M E Dietz H C Lessons on the pathogenesis of aneurysm from heritable conditions Nature 2011 473(7347):308 316 21593863 10.1038/nature10145 PMC3622871

[45] Gomez D Al Haj Zen A Borges L F Syndromic and non-syndromic aneurysms of the human ascending aorta share activation of the Smad2 pathway J Pathol 2009 218 01 131 142 19224541 10.1002/path.2516

[46] Borges L F Gomez D Quintana M Fibrinolytic activity is associated with presence of cystic medial degeneration in aneurysms of the ascending aorta Histopathology 2010 57 06 917 932 21166705 10.1111/j.1365-2559.2010.03719.x

[47] Gomez D Coyet A Ollivier V Epigenetic control of vascular smooth muscle cells in Marfan and non-Marfan thoracic aortic aneurysms Cardiovasc Res 2011 89 02 446 456 20829218 10.1093/cvr/cvq291 PMC3020128

[48] Gomez D Kessler K Michel J B Vranckx R Modifications of chromatin dynamics control Smad2 pathway activation in aneurysmal smooth muscle cells Circ Res 2013 113 07 881 890 23825360 10.1161/CIRCRESAHA.113.301989

[49] Gomez D Kessler K Borges L F Smad2-dependent protease nexin-1 overexpression differentiates chronic aneurysms from acute dissections of human ascending aorta Arterioscler Thromb Vasc Biol 2013 33 09 2222 2232 23814118 10.1161/ATVBAHA.113.301327

[50] Shah A A Gregory S G Krupp D Epigenetic profiling identifies novel genes for ascending aortic aneurysm formation with bicuspid aortic valves Heart Surg Forum 2015 18 04 E134 E139 26334848 10.1532/hsf.1247

[51] Liao M Zou S Weng J A microRNA profile comparison between thoracic aortic dissection and normal thoracic aorta indicates the potential role of microRNAs in contributing to thoracic aortic dissection pathogenesis J Vasc Surg 2011 53 05 1341 1.349E6 21334170 10.1016/j.jvs.2010.11.113

[52] Jones J A Stroud R E O'Quinn E C Selective microRNA suppression in human thoracic aneurysms: relationship of miR-29a to aortic size and proteolytic induction Circ Cardiovasc Genet 2011 4 06 605 613 22010139 10.1161/CIRCGENETICS.111.960419 PMC3246193

[53] Patuzzo C Pasquali A Malerba G A preliminary microRNA analysis of non syndromic thoracic aortic aneurysms Balkan J Med Genet 2012 15(15, Suppl):51 55 10.2478/v10034-012-0019-6 PMC3776682 24052744

[54] Venkatesh P Phillippi J Chukkapalli S Aneurysm-specific miR-221 and miR-146a participates in human thoracic and abdominal aortic aneurysms Int J Mol Sci 2017 18 04 184 10.3390/ijms18040875 PMC5412456 28425970

[55] Premakumari V Chukkapalli S Rivera M Microrna expression signature in human thoracic and abdominal aortic aneurysms Atherosclerosis 2014 235 02 e130

[56] Boon R A Dimmeler S MicroRNAs and aneurysm formation Trends Cardiovasc Med 2011 21 06 172 177 22814425 10.1016/j.tcm.2012.05.005

[57] Boon R A Seeger T Heydt S MicroRNA-29 in aortic dilation: implications for aneurysm formation Circ Res 2011 109 10 1115 1119 21903938 10.1161/CIRCRESAHA.111.255737

[58] Merk D R Chin J T Dake B A miR-29b participates in early aneurysm development in Marfan syndrome Circ Res 2012 110 02 312 324 22116819 10.1161/CIRCRESAHA.111.253740

[59] Chen K C Wang Y S Hu C Y OxLDL up-regulates microRNA-29b, leading to epigenetic modifications of MMP-2/MMP-9 genes: a novel mechanism for cardiovascular diseases FASEB J 2011 25 05 1718 1728 21266537 10.1096/fj.10-174904

[60] Mott J L Kobayashi S Bronk S F Gores G J mir-29 regulates Mcl-1 protein expression and apoptosis Oncogene 2007 26 42 6133 6140 17404574 10.1038/sj.onc.1210436 PMC2432524

[61] Cushing L Costinean S Xu W Disruption of miR-29 leads to aberrant differentiation of smooth muscle cells selectively associated with distal lung vasculature PLoS Genet 2015 11 05 e1005238 26020233 10.1371/journal.pgen.1005238 PMC4447351

[62] Deaton R A Gan Q Owens G K Sp1-dependent activation of KLF4 is required for PDGF-BB-induced phenotypic modulation of smooth muscle Am J Physiol Heart Circ Physiol 2009 296 04 H1027 H1037 19168719 10.1152/ajpheart.01230.2008 PMC2670704

[63] van Rooij E Sutherland L B Thatcher J E Dysregulation of microRNAs after myocardial infarction reveals a role of miR-29 in cardiac fibrosis Proc Natl Acad Sci U S A 2008 105 35 13027 13032 18723672 10.1073/pnas.0805038105 PMC2529064

[64] Du Y Gao C Liu Z Upregulation of a disintegrin and metalloproteinase with thrombospondin motifs-7 by miR-29 repression mediates vascular smooth muscle calcification Arterioscler Thromb Vasc Biol 2012 32 11 2580 2588 22995515 10.1161/ATVBAHA.112.300206

[65] Cordes K R Sheehy N T White M P miR-145 and miR-143 regulate smooth muscle cell fate and plasticity Nature 2009 460(7256):705 710 19578358 10.1038/nature08195 PMC2769203

[66] Pei H Tian C Sun X Overexpression of microRNA-145 promotes ascending aortic aneurysm media remodeling through TGF-β1 Eur J Vasc Endovasc Surg 2015 49 01 52 59 25465469 10.1016/j.ejvs.2014.10.018

[67] Elia L Quintavalle M Zhang J The knockout of miR-143 and -145 alters smooth muscle cell maintenance and vascular homeostasis in mice: correlates with human disease Cell Death Differ 2009 16 12 1590 1598 19816508 10.1038/cdd.2009.153 PMC3014107

[68] Cheng Y Liu X Yang J MicroRNA-145, a novel smooth muscle cell phenotypic marker and modulator, controls vascular neointimal lesion formation Circ Res 2009 105 02 158 166 19542014 10.1161/CIRCRESAHA.109.197517 PMC2728297

[69] Hergenreider E Heydt S Tréguer K Atheroprotective communication between endothelial cells and smooth muscle cells through miRNAs Nat Cell Biol 2012 14 03 249 256 22327366 10.1038/ncb2441

[70] Chen J Yin H Jiang Y Induction of microRNA-1 by myocardin in smooth muscle cells inhibits cell proliferation Arterioscler Thromb Vasc Biol 2011 31 02 368 375 21051663 10.1161/ATVBAHA.110.218149 PMC3207238

[71] Xie C Huang H Sun X MicroRNA-1 regulates smooth muscle cell differentiation by repressing Kruppel-like factor 4 Stem Cells Dev 2011 20 02 205 210 20799856 10.1089/scd.2010.0283 PMC3128754

[72] Torella D Iaconetti C Catalucci D MicroRNA-133 controls vascular smooth muscle cell phenotypic switch in vitro and vascular remodeling in vivo Circ Res 2011 109 08 880 893 21852550 10.1161/CIRCRESAHA.111.240150

[73] Li P Zhu N Yi B MicroRNA-663 regulates human vascular smooth muscle cell phenotypic switch and vascular neointimal formation Circ Res 2013 113 10 1117 1127 24014830 10.1161/CIRCRESAHA.113.301306 PMC4537615

[74] Merlet E Atassi F Motiani R K miR-424/322 regulates vascular smooth muscle cell phenotype and neointimal formation in the rat Cardiovasc Res 2013 98 03 458 468 23447642 10.1093/cvr/cvt045 PMC3656613

[75] Wang Y S Wang H Y Liao Y C MicroRNA-195 regulates vascular smooth muscle cell phenotype and prevents neointimal formation Cardiovasc Res 2012 95 04 517 526 22802111 10.1093/cvr/cvs223

[76] Li P Liu Y Yi B MicroRNA-638 is highly expressed in human vascular smooth muscle cells and inhibits PDGF-BB-induced cell proliferation and migration through targeting orphan nuclear receptor NOR1 Cardiovasc Res 2013 99 01 185 193 23554459 10.1093/cvr/cvt082 PMC3687750

[77] Liu X Cheng Y Yang J Xu L Zhang C Cell-specific effects of miR-221/222 in vessels: molecular mechanism and therapeutic application J Mol Cell Cardiol 2012 52 01 245 255 22138289 10.1016/j.yjmcc.2011.11.008 PMC3664545

[78] Leeper N J Raiesdana A Kojima Y MicroRNA-26a is a novel regulator of vascular smooth muscle cell function J Cell Physiol 2011 226 04 1035 1043 20857419 10.1002/jcp.22422 PMC3108574

[79] Dong S Xiong W Yuan J Li J Liu J Xu X MiRNA-146a regulates the maturation and differentiation of vascular smooth muscle cells by targeting NF-κB expression Mol Med Rep 2013 8 02 407 412 23784108 10.3892/mmr.2013.1538

[80] Zhang J Zhao F Yu X Lu X Zheng G MicroRNA-155 modulates the proliferation of vascular smooth muscle cells by targeting endothelial nitric oxide synthase Int J Mol Med 2015 35 06 1708 1714 25872580 10.3892/ijmm.2015.2181

[81] Liu X Cheng Y Chen X Yang J Xu L Zhang C MicroRNA-31 regulated by the extracellular regulated kinase is involved in vascular smooth muscle cell growth via large tumor suppressor homolog 2 J Biol Chem 2011 286 49 42371 42380 22020941 10.1074/jbc.M111.261065 PMC3234904

[82] Wang J Yan C H Li Y MicroRNA-31 controls phenotypic modulation of human vascular smooth muscle cells by regulating its target gene cellular repressor of E1A-stimulated genes Exp Cell Res 2013 319 08 1165 1175 23518389 10.1016/j.yexcr.2013.03.010

[83] Li T J Chen Y L Gua C J Xue S J Ma S M Li X D MicroRNA 181b promotes vascular smooth muscle cells proliferation through activation of PI3K and MAPK pathways Int J Clin Exp Pathol 2015 8 09 10375 10384 26617745 PMC4637560

[84] Davis B N Hilyard A C Lagna G Hata A SMAD proteins control DROSHA-mediated microRNA maturation Nature 2008 454(7200):56 61 18548003 10.1038/nature07086 PMC2653422

[85] Ji R Cheng Y Yue J MicroRNA expression signature and antisense-mediated depletion reveal an essential role of microRNA in vascular neointimal lesion formation Circ Res 2007 100 11 1579 1588 17478730 10.1161/CIRCRESAHA.106.141986

[86] Yang J Chen L Ding J MicroRNA-24 inhibits high glucose-induced vascular smooth muscle cell proliferation and migration by targeting HMGB1 Gene 2016 586 02 268 273 27085480 10.1016/j.gene.2016.04.027

[87] Fiedler J Stöhr A Gupta S K Functional microRNA library screening identifies the hypoxamir miR-24 as a potent regulator of smooth muscle cell proliferation and vascularization Antioxid Redox Signal 2014 21 08 1167 1176 24063572 10.1089/ars.2013.5418 PMC4142777

[88] Wanhainen A Mani K Vorkapic E Screening of circulating microRNA biomarkers for prevalence of abdominal aortic aneurysm and aneurysm growth Atherosclerosis 2017 256 82 88 27993388 10.1016/j.atherosclerosis.2016.11.007

[89] Yu B Liu L Sun H Chen Y Long noncoding RNA AK056155 involved in the development of Loeys-Dietz syndrome through AKT/PI3K signaling pathway Int J Clin Exp Pathol 2015 8 09 10768 10775 26617788 PMC4637603

[90] Zhao Y Feng G Wang Y Yue Y Zhao W Regulation of apoptosis by long non-coding RNA HIF1A-AS1 in VSMCs: implications for TAA pathogenesis Int J Clin Exp Pathol 2014 7 11 7643 7652 25550800 PMC4270571

[91] Wang S Zhang X Yuan Y BRG1 expression is increased in thoracic aortic aneurysms and regulates proliferation and apoptosis of vascular smooth muscle cells through the long non-coding RNA HIF1A-AS1 in vitro Eur J Cardiothorac Surg 2015 47 03 439 446 24875884 10.1093/ejcts/ezu215

[92] Wang Y N Shan K Yao M D Long noncoding RNA-GAS5: a novel regulator of hypertension-induced vascular remodeling Hypertension 2016 68 03 736 748 27432865 10.1161/HYPERTENSIONAHA.116.07259

[93] Shan K Jiang Q Wang X Q Role of long non-coding RNA-RNCR3 in atherosclerosis-related vascular dysfunction Cell Death Dis 2016 7 06 e2248 27253412 10.1038/cddis.2016.145 PMC5143375

[94] Congrains A Kamide K Katsuya T CVD-associated non-coding RNA, ANRIL, modulates expression of atherogenic pathways in VSMC Biochem Biophys Res Commun 2012 419 04 612 616 22382030 10.1016/j.bbrc.2012.02.050

[95] Liu W Liu X Luo M dNK derived IFN-γ mediates VSMC migration and apoptosis via the induction of LncRNA MEG3: A role in uterovascular transformation Placenta 2017 50 32 39 28161059 10.1016/j.placenta.2016.12.023

[96] Lv J Wang L Zhang J Long noncoding RNA H19-derived miR-675 aggravates restenosis by targeting PTEN Biochem Biophys Res Commun 2017 S0006-291X(17)30011-6 10.1016/j.bbrc.2017.01.011 28063931

[97] Wu G Cai J Han Y LincRNA-p21 regulates neointima formation, vascular smooth muscle cell proliferation, apoptosis, and atherosclerosis by enhancing p53 activity Circulation 2014 130 17 1452 1465 25156994 10.1161/CIRCULATIONAHA.114.011675 PMC4244705

[98] Bell R D Long X Lin M Identification and initial functional characterization of a human vascular cell-enriched long noncoding RNA Arterioscler Thromb Vasc Biol 2014 34 06 1249 1259 24578380 10.1161/ATVBAHA.114.303240 PMC4024079

[99] Ingber D E Tensegrity II. How structural networks influence cellular information processing networks J Cell Sci 2003 116(Pt 8, 116Pt 8):1397 1408 12640025 10.1242/jcs.00360

[100] Wang N Tytell J D Ingber D E Mechanotransduction at a distance: mechanically coupling the extracellular matrix with the nucleus Nat Rev Mol Cell Biol 2009 10 01 75 82 19197334 10.1038/nrm2594

[101] Isermann P Lammerding J Nuclear mechanics and mechanotransduction in health and disease Curr Biol 2013 23 24 R1113 R1121 24355792 10.1016/j.cub.2013.11.009 PMC3883624

[102] Sun S G Zheng B Han M miR-146a and Krüppel-like factor 4 form a feedback loop to participate in vascular smooth muscle cell proliferation EMBO Rep 2011 12 01 56 62 21109779 10.1038/embor.2010.172 PMC3024123

[103] Di Gregoli K Mohamad Anuar N N Bianco R MicroRNA-181b controls atherosclerosis and aneurysms through regulation of TIMP-3 and elastin Circ Res 2017 120 01 49 65 27756793 10.1161/CIRCRESAHA.116.309321 PMC5214094

[104] Kee H J Kim G R Cho S N miR-18a-5p microRNA increases vascular smooth muscle cell differentiation by downregulating Syndecan4 Korean Circ J 2014 44 04 255 263 25089138 10.4070/kcj.2014.44.4.255 PMC4117847

[105] Li J Zhao L He X Yang T Yang K MiR-21 inhibits c-Ski signaling to promote the proliferation of rat vascular smooth muscle cells Cell Signal 2014 26 04 724 729 24388835 10.1016/j.cellsig.2013.12.013

[106] Yang G Pei Y Cao Q Wang R MicroRNA-21 represses human cystathionine gamma-lyase expression by targeting at specificity protein-1 in smooth muscle cells J Cell Physiol 2012 227 09 3192 3200 22034194 10.1002/jcp.24006

[107] le Sage C Nagel R Egan D A Regulation of the p27(Kip1) tumor suppressor by miR-221 and miR-222 promotes cancer cell proliferation EMBO J 2007 26 15 3699 3708 17627278 10.1038/sj.emboj.7601790 PMC1949005

[108] Davis B N Hilyard A C Nguyen P H Lagna G Hata A Induction of microRNA-221 by platelet-derived growth factor signaling is critical for modulation of vascular smooth muscle phenotype J Biol Chem 2009 284 06 3728 3738 19088079 10.1074/jbc.M808788200 PMC2635044

[109] Zhu X F Shan Z Ma J Y Investigating the role of the posttranscriptional gene regulator MiR-24- 3p in the proliferation, migration and apoptosis of human arterial smooth muscle cells in arteriosclerosis obliterans Cell Physiol Biochem 2015 36 04 1359 1370 26159387 10.1159/000430302

